# Europe must act: rethinking global health preparedness without the United States

**DOI:** 10.1093/eurpub/ckag098

**Published:** 2026-06-17

**Authors:** Anja Schreijer, Tiago Correia, Martin McKee, Tit Albreht, Aura Timen, Maria Ganczak, Tony Holohan

**Affiliations:** Pandemic and Disaster Preparedness Center, Erasmus Medical Center, Rotterdam, the Netherlands; European Public Health Association, Utrecht, the Netherlands; Associate Laboratory in Translation and Innovation Towards Global Health, LA-REAL, Global Health and Tropical Medicine, GHTM, Instituto de Higiene e Medicina Tropical, IHMT, Universidade Nova de Lisboa, UNL, Lisbon, Portugal; WHO Collaborating Center for Health Workforce Policy and Planning, Instituto de Higiene e Medicina Tropical, IHMT, Universidade Nova de Lisboa, UNL, Lisbon, Portugal; European Public Health, London School of Hygiene & Tropical Medicine, London, United Kingdom; Department of Public Health, Faculty of Medicine, University of Ljubljana, Ljubljana, Slovenia; European Public Health Association, Utrecht, the Netherlands; Primary and Community Care, Nijmegen University Medical Center, Nijmegen, the Netherlands; European Public Health Association, Utrecht, the Netherlands; Department of Infectious Diseases, Institute of Medical Sciences, Collegium Medicum, University of Zielona Góra, Zielona Góra, Poland; WHO Collaborating Centre on One Health, College of Health and Agricultural Sciences, University College Dublin, Ireland

In a world sensitized by COVID-19 to the risks of unusual infectious diseases, news of outbreaks of Hantavirus and the Bundibugyo strain of Ebola attracted widespread media attention [1, 2]. The circumstances were very different, but they should not be seen as isolated events. Rather, they are signals of a deeper shift in global health, where vulnerabilities are shaped as much by politics as by pathogens themselves. Multilateral instruments, such as the International Health Regulations (IHR), are being challenged [3].

Much has been written about the lessons from the COVID-19 pandemic. There were remarkable successes, such as the unprecedentedly rapid development of new vaccines, but also many failures. A series of reports called for measures to strengthen national, regional, and global preparedness and responses to future pandemic threats [4, 5]. Some have been implemented, but many have not. The failure to reach consensus on the Pathogen Access and Benefit Sharing annex to the WHO Pandemic Agreement reveals how far the international community remains from the solidarity it repeatedly invokes [6].

Worse, in some respects, things are moving backwards. The multilateral system that underpinned global action on health since the 1940s is under threat. The United States has withdrawn from the WHO, taking its funding with it and seriously weakening the organization [7]. It has blocked its officials from official communication with the WHO, although informal, unsanctioned contacts continue [8]. It has also withdrawn from mechanisms that address the determinants of health, such as the Paris Climate Agreement [9]. It has attacked the organizations that uphold international law, most notably the International Criminal Court [10]. It has slashed its development assistance, leaving major gaps in global surveillance and response. At the same time, within its own borders, it has dismantled crucial elements of its health defenses and weakened trust in vaccines, leaving its population at risk from vaccine-preventable diseases and emerging infections.

These developments mark a profound shift in global health governance, making it essential for the international community to fill the vacuum left by US disengagement. Some progress is evident, with initiatives such as the Accra Reset creating a mechanism for African countries to assume greater responsibility, strengthen their global voice, and mobilize resources aligned with their priorities [11]. But serious gaps remain.

As COVID-19 demonstrated, inaction is now a strategic risk in a fragmented world. Without stronger leadership, other actors will shape global health norms and priorities, potentially weakening equity and multilateral cooperation [12]. This is especially critical as interconnected threats, spanning human, animal, plant, and environmental health through climate change, biodiversity loss, and conflict, outpace current preparedness frameworks, increasing the risk that outbreaks escalate and spread [11].

Europe has a particular responsibility in global health, given its financial, regulatory, and scientific capacity, as well as its responsibilities to address the legacy of colonialism. Yet it remains constrained by limited political coherence, internal divisions [13], and its current global health strategy lacks ambition [14].

So what, in practice, does this mean for Europe? A credible strategy must recognize that health is no longer a purely technical domain but a core element of geopolitical stability. In a context of weakening multilateralism, Europe must step forward to reinforce and renew global cooperation, providing sustained political and financial support to institutions such as the WHO and taking a leading role in taking forward, on an equitable basis, the Pandemic Agreement. The European Commission’s newly launched Global Health Resilience Initiative offers a vision for strengthening preparedness, improving coordination, and supporting resilient health systems [15], but it will require moving beyond a rhetorical commitment to equity and embedding it in practice through trust-building measures, including fair benefit-sharing and technology transfer. It must also align closely with WHO and the International Health Regulations.

Europe must continue to invest in global public goods that underpin preparedness: robust surveillance systems, laboratory networks, and a well-trained workforce capable of responding to emerging threats. Many of these resources previously depended on US funding. These investments must be matched by a more strategic approach to innovation that corrects the persistent failure of markets to deliver vaccines and treatments for neglected diseases through long-term, coordinated research funding [16]. Horizon Europe should prioritize flexible vaccine platforms, including mRNA technologies, as well as broad-spectrum antivirals, diagnostics, manufacturing capacity and adaptive trial networks [17].

Equity must underpin preparedness, ensuring access to countermeasures, strengthening manufacturing in low- and middle-income countries, and fostering genuinely collaborative partnerships. Europe must also tackle internal inequalities, which allow infections to persist and spread, threatening all.

A European global health strategy must align health with climate, development, and foreign policy, recognizing their interdependence. This can help rebuild a more coherent and equitable global health architecture protecting both vulnerable populations and European citizens. Stronger academic and global research collaboration will enhance preparedness. Evidence shows urgent health needs shape research trajectories, as in responses to influenza, Zika, and SARS. Expanding research to understand emerging pathogens, develop targeted treatments, and implement effective controls would enable Europe to lead by example [3].

Preparedness must be driven by political action and not treated as a purely technical exercise. Surveillance and medical countermeasures are essential but insufficient. Effective preparedness depends on solidarity, strong governance, and fair distribution of power, resources, and technologies. Without these, technical capacities may reinforce inequalities, leaving global systems fragile. Resilience also requires public literacy and agency, enabling people to understand risks, trust institutions, and act.

In a shifting global health order, equity must be foundational. Without fair access to resources, technologies, and decision-making, preparedness will fail; without decisive European leadership, progress will stall. Europe faces a clear choice: lead in rebuilding a cooperative system or accept growing fragmentation, deeper inequalities, and reduced security.

Conflict of interest: None declared.
